# Self-assembled graphene-based microfibers with eclectic optical properties

**DOI:** 10.1038/s41598-021-84940-0

**Published:** 2021-03-09

**Authors:** Mahdi Ghamsari, Tayyebeh Madrakian, Abbas Afkhami, Mazaher Ahmadi

**Affiliations:** Faculty of Chemistry, Bu-Ali Sina University, Hamedan, 6517838695 Iran

**Keywords:** Graphene, Synthesis and processing

## Abstract

The construction of graphene-based microfibers with reinforced mechanical and electrical properties has been the subject of numerous researches in recent years. However, the fabrication of graphene-based fibers with remarkable optical features still remains a challenge and has not been addressed so far. This paper aims to report a series of flexible self-assembled fibers, synthesized through a few-minute sonication of thermally oxidized graphene oxide nanosheets, so-called Nanoporous Over-Oxidized Graphene (NOG), in an acidic medium. These free-standing glassy fibers were classified into four distinct morphological structures and displayed a collection of intriguing optical properties comprising high transparency, strong birefringence, fixed body colorations (e.g. colorless, blue, green, and red), tunable interference marginal colorations, UV–visible-near IR fluorescence, and upconversion emissions. Moreover, they exhibited high chemical stability in strongly acidic, basic, and oxidizing media. The foregoing notable attributes introduce the NOG fiber as a promising candidate both for the construction of graphene-based photoluminescent textiles and the development of a wide variety of optical applications.

## Introduction

Graphene-based nanomaterials, constructed of one-atom-thick two-dimensional layers of *sp*^2^-bonded carbons, have already shown a great promise in advanced applications on account of their fascinating physiochemical properties ranging from giant intrinsic carrier mobility^1^, chiral quantum Hall effects^2^ to high thermal conductivity^3^ and extraordinary mechanical stiffness^4^. However, the acquisition of these attractive features in practice is highly demanding and lots of researchers have exerted themselves to bridge the gap between theory and practice. One possible route to harnessing these properties in different applications would be to incorporate the nanosheets into macroscopic scaffolds. Assembly of graphene-based nanosheets into assorted macroscopic architectures such as fibers^5^, sponges^6^, beads^7^, and membranes^8^ has been the subject of intense researches in recent years. In fact, the foregoing strategy not only holds the promise for preserving beneficial aspects of the nanosheets like high specific surface area and surface functionalities but also eliminates the deployment adversity of the powder for specific applications such as energy storage devices^9^, catalysis^10^, environmental applications^11^ and so on. In particular, graphene microfibers (GMFs) have been exploited in a wide variety of applications ranging from electronic textiles^12^ and flexible supercapacitors^13^ to environmental remediation^11^. To date, lots of efforts have been intensively focused on the preparation of GMFs by various methods including wet-spinning, dry-spinning, and plasticization spinning of GO liquid crystals^5,14–16^, baking of GO dispersion in a sealed pipeline^17^, scrolling of GO films^18^, and microfluidic 3D printing technology^19,20^. However, the vast majority of attention have been paid to reinforcing their mechanical and electrical properties^20^ and there has been already no previous evidence of GMFs with notable optical features. Therefore, synthesis of GMFs with focus on their optical properties is still challenging and could extend the GMFs versatility to optics and photonics.

**Figure 1:**
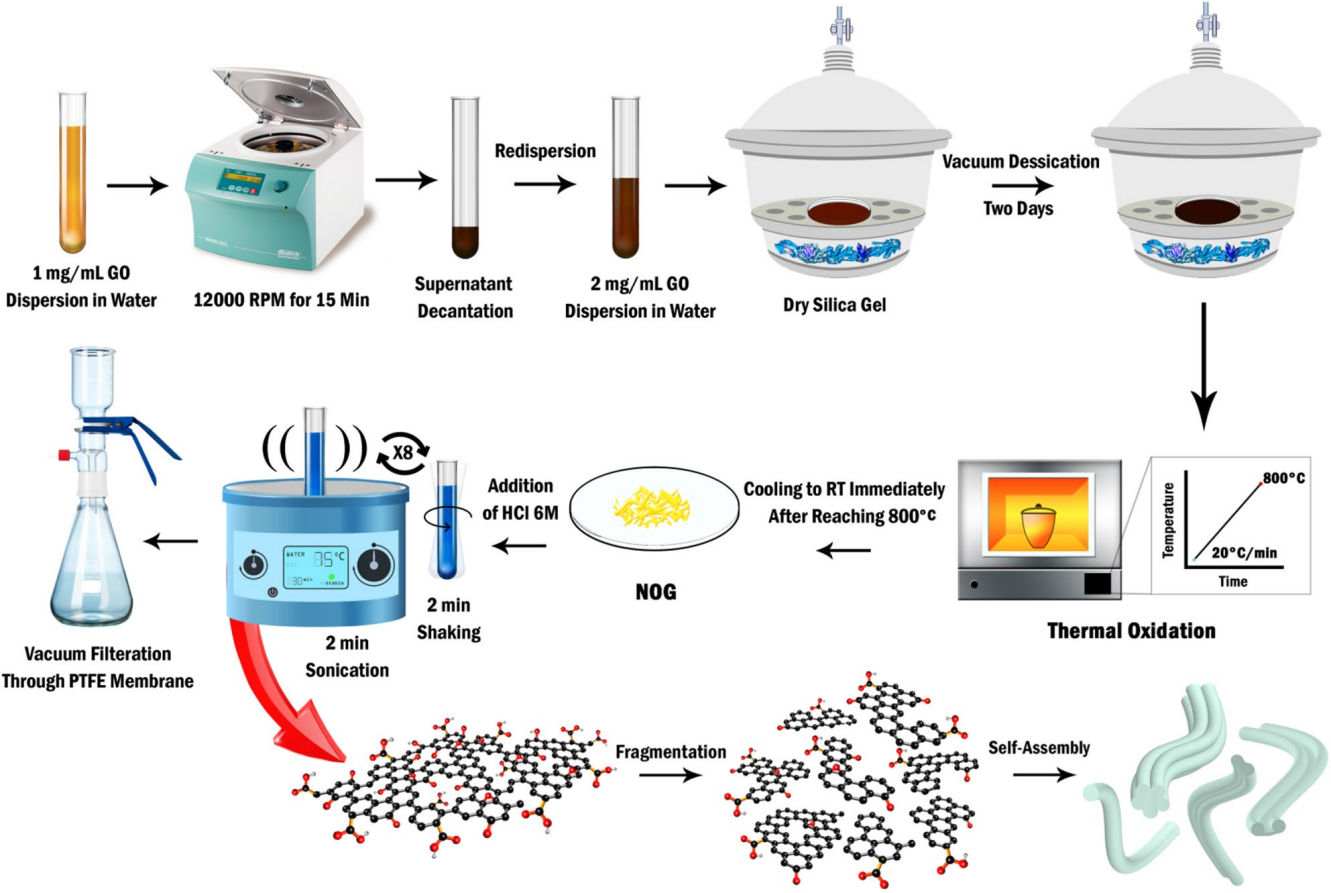
Schematic illustration of the synthetic procedure of NOG nanosheets and the self-assembled NOG microfibers.

Selection of a potential candidate for meeting the above challenge among a multitude of reported graphene-based materials would be quite crucial. In particular, several studies have been published in the past few years on various optical aspects of graphene and graphene oxide nanosheets including transparency^21^, photoluminescence (PL)^22^, and coloration^23–27^; however, none of these features have been displayed in the resulting fibers so far. Herein, a new type of nanoporous graphene which was synthesized through thermal oxidation of orderly graphene oxide sediments (OGOS) up to 800 °C showed a great promise in this regard. In fact, this unique material, so-called nanoporous over-oxidized graphene (NOG), showed remarkable optical properties including high transparency, UV–visible-near IR fluorescence, as well as upconversion luminescence (UL). Besides, NOG nanosheets exhibited great hydrophilicity owing to their high oxidation degree (C/O:1.67) and were well dispersed in water to form a clear and colorless dispersion. Nevertheless, although acidification of the dispersion to pH > 2 only led to unshaped flocculated particles, lower pH values triggered a self-assembly process, giving rise to some flexible transparent fibers whose number and sizes increase significantly by sonication in the ultrasonic bath (Fig. 1). Further investigations uncovered a series of colorful glassy GMFs exhibiting a collection of intriguing optical properties, expected to be advantageous for extension of GMFs applicability to optical areas.

In this paper, at first, in an effort to shed a light upon the role of thermal oxidation in the synthesis of NOG nanosheets, the changes of OGOS during this process were inspected by means of TGA, TEM, FE-SEM, XPS, and UV/visible absorption analyses. Then, the transformation mechanism of NOG nanosheets into the self-assembled microfibers together with the characterization of the resulting fibers were discussed on the basis of optical microscopy, polarized-optical microscopy, epifluorescence microscopy, confocal laser scanning microscopy, FE-SEM, TEM, and DLS spectrophotometry findings.

## Results and discussion

### Thermal oxidation of OGOS

Two OGOS samples were analyzed by TGA in air and nitrogen atmospheres and the occurence of thermal oxidation in annealing process was proved by comparison of the two curves in temperatures above 500 °C. At this point, although OGOS underwent a sharp mass-loss as a result of oxidation in the air atmosphere, it persisted with the reduction process with a mass-loss rate of (~ 0.74%/min) in N_2_ atmosphere (Fig. S1).

**Figure 2:**
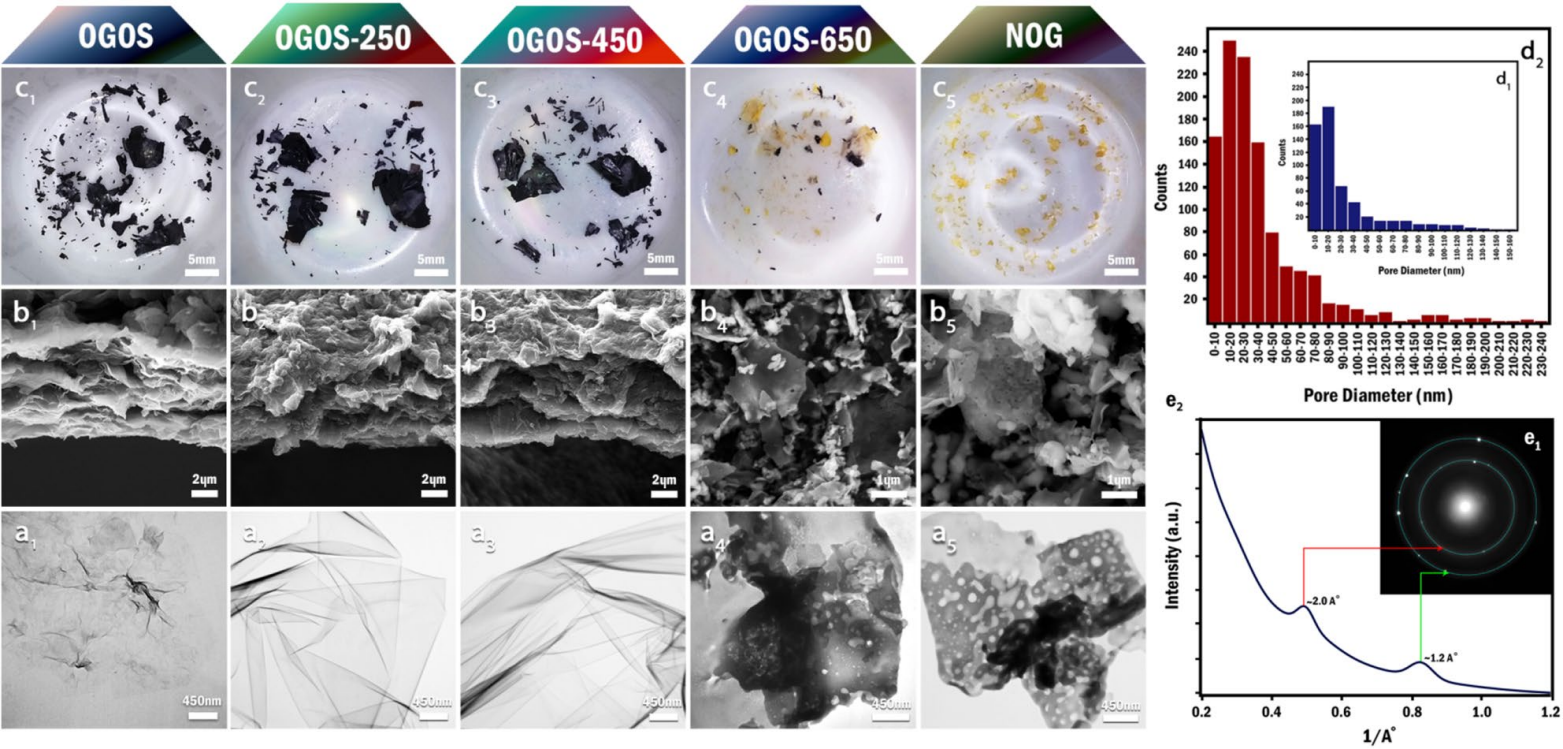
Inspection of OGOS alterations during the annealing in air atmosphere, OGOS-250, OGOS-450, OGOS-650, and NOG are representatives of samples heated up to 250 °C, 450 °C, 650 °C, and 800 °C respectively. (**a**_**1**_**–a**_**5**_) TEM images of the nanosheets after dispersing in deionized water. (**b**_**1**_**–b**_**3**_) Cross-Section FE-SEM images of OGOS, OGOS-250, and OGOS-450 samples. (**b**_**4**_**–b**_**5**_) FE-SEM images of powdered OGOS-650 and NOG samples. (**c**_**1**_**–c**_**5**_) Photographs of the samples in the crucible, immediately after cooling down to room temperature. (**d**_**1**_**–d**_**2**_) Histogram of the nanopores' diameter size for OGOS-650 and NOG respectively. (**e**_**1**_**–e**_**2**_) SAED pattern of NOG nanosheets and the corresponding diffraction intensity profile.

TEM and FE-SEM investigations were carried out to follow the morphological changes of OGOS in this process. Firstly, it was revealed that the initial OGOS sample had been constituted of both single and few-layered GO nanosheets in a well-ordered manner (Fig. 2a1,b1). Secondly, following the temperature raising up to 450 °C, although the evolution of CO_2_ and CO gases slightly disrupted the order of superficial nanosheets, the arrangement of beneath layers remained intact (Fig. 2b1–b3). Moreover, no signs of nanopores were pinpointed up to 450 °C (Fig. 2a2,a3). From then on, the elevation of temperature not only gave rise to the etching of nanosheets but also broke them into smaller pieces and diminished their lateral dimentions from ~ 10 µm in OGOS-450 to ~ 400–600 nm in NOG (Fig. 2a4,b4). Furthermore, distribution diagrams of the nanopores' diameters for two samples of OGOS-650 and NOG disclosed that raising of temperature, firstly, had boosted the nanopores' density and secondly, had increased the average diameter from 26 to 36 nm and the diameters' range from 3–160 nm to 3–240 nm (Fig. 2d1,d2). More explicitly, at these high temperatures, oxygen not only attacks the basal plan of the nanosheets to form new nanopores but also burns the edges and finally merges some pores to increase the average diameter (Fig. 2a5,b5). The raising of the temperature from 650 to 800 °C, engendered less amount of the final product; however, pursuant to the photographs, (Fig. 2c1–c5), utterly transparent material cannot be acquired unless the temperature reaches 800 °C. The SAED patterns obtained from different regions of NOG nanosheets showed two broad diffuse rings at ~ 1.2 Å, ~ 2.0 Å that are consistent with the reported values for the amorphous carbon materials in the literature (Fig. 2e1–e2)^28^.

**Figure 3:**
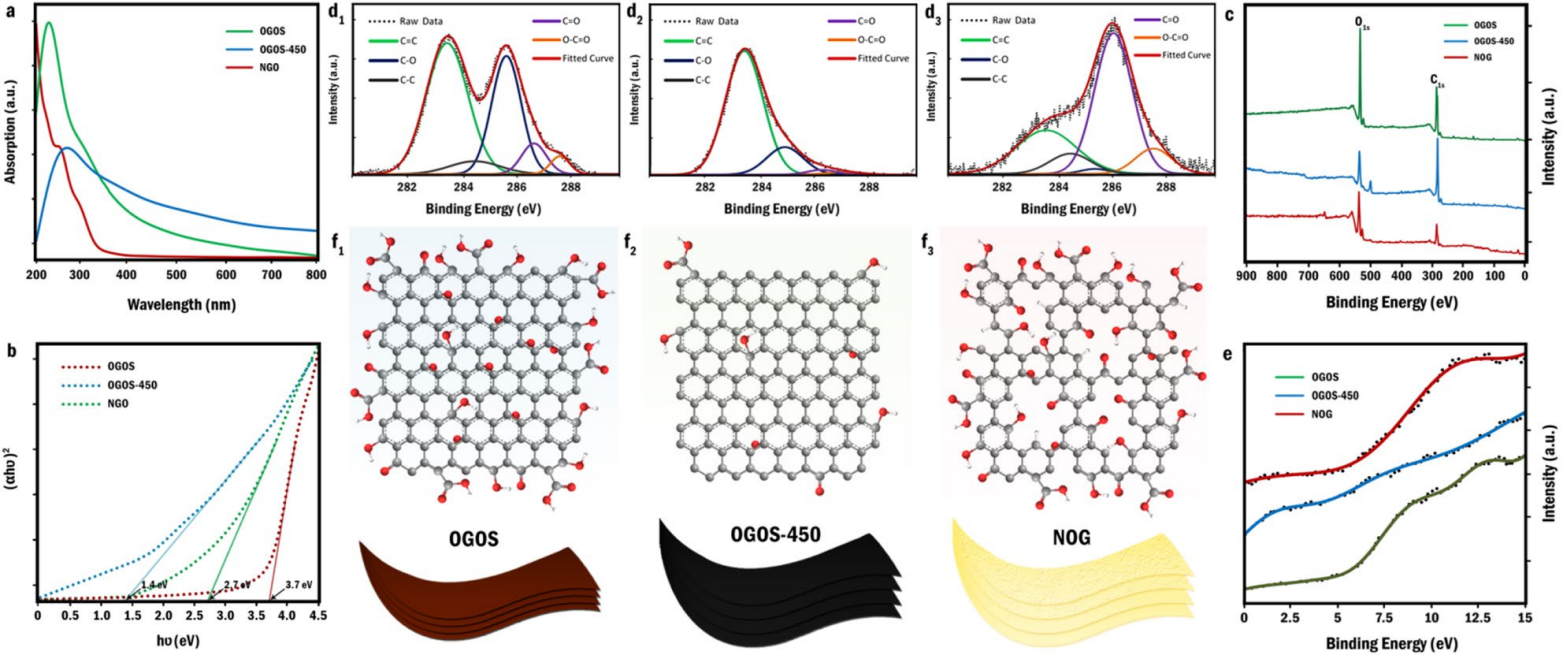
Ultraviolet–visible (UV–Vis) spectroscopy and X-ray photoelectron spectroscopy analyses, (**a**) UV/Visible spectra of (1 mg/mL) dispersions of OGOS, OGOS-450, and NOG samples (**b**) Tauc plots derived from UV/Visible spectra for determination of the samples' band gaps (**c**) Wide scan XPS spectra of the samples (**d**_**1**_**–d**_**3**_) Deconvoluted high-resolution XPS C1s spectra of OGOS, OGOS-450, and NOG samples respectively. (**e**) XPS valence band spectra of the samples, and (**f**_**1**_**–f**_**3**_) Schematic illustration of OGOS chemical structure alteration during the thermal oxidation.

The changes occurred in band gap and electronic transitions of OGOS as a result of annealing process were examined through UV–Visible spectroscopy (Fig. 3a,b). The absorption spectra of OGOS and OGOS-450, as its thermally reduced form, were in line with the reported spectra of GO and rGO respectively^29–31^. Following the thermal oxidation, firstly, the overall absorption of OGOS and OGOS-450 in the visible region was considerably eliminated and secondly, the strong absorption band at 268 nm was replaced with two consecutive shoulders at about 230 and 276 nm which the first one is most likely attributed to π–π* transitions of *sp*^2^ hybridized carbons and the latter seems to be the peer of the GO absorption shoulder for n–π* transitions of C=O bonds. According to Tauc plots, the optical band gap of the initial OGOS was determined 2.7 eV which is consistent with the previous literature reports^30^. This gap diminished subsequently to 1.4 eV in OGOS-450 and then opened up to 3.7 eV in NOG. Such a large band gap rationalizes the transparency of NOG nanosheets.

In order to elucidate the chemical structures of the foregoing samples, XPS analyses were carried out. According to wide scan spectra, the C/O ratio of OGOS (2.27) initially increased to 3.48 in OGOS-450 owing to the thermal reduction process and, subsequently, dropped to the value of 1.67 in NOG^30,32^. (Fig. 3c) These variations were scrutinized by deconvolution and quantification of high-resolution C_1s_ core-level photoemission spectra of each samples (Fig. 3d1–d3) (Table 1). According to OGOS-450 spectrum, the contribution of all oxygen-containing functionalities was trivialized as a consequence of the thermal reduction process. All the same, in the case of NOG, high-temperature etching along with oxidation of most of the other C=C bonds to oxygen-containing functional groups accounts for the significant diminution of C=C percentage from 79.2 to 26.7%^33^. Additionally, depletion of C–O species coupled with the remarkable escalation of carbonyl percentage within 51.93% and increment of carboxyl content up to 7.79% in thermal oxidation process are most likely attributed to the fact that at the elevated temperatures, oxygen is prone to incorporate itself into graphene lattice both to form epoxides on the basal plane (energy barrier ~ 0.3 eV) and semiquinones, carbonyls, lactones, carboxylic acids, and ethers at defects^34–36^. Moreover, while the thermal reduction gives rise to the recovery of conjugated *sp*^2^ network along with the diminution of *sp*^3^ carbons in OGOS-450^37,38^, the introduction of numerous defects by means of thermal oxidation process accounts for the recreation of sp^3^-hybridized carbons in NOG^39–42^. XPS at low binding energies can also be exploited to probe the electron density of the valence band on the surface of the samples (Fig. 3e). In contrast to OGOS, as the initial precursor exhibiting a valence band far away from the Fermi level (E_F_), the nearest peak found in OGOS-450 almost coincides on Fermi level which such substantial shift to lower binding energies (− 9.75 eV) can be mainly attributed to band gap decrement as a consequence of thermal reduction. Most importantly, the valence band peak of NOG appeared much farther away from OGOS peak (11.5 eV) which well-substantiates the role of thermal oxidation in band-gap opening of GO. Figure 3f1–f3 shows the schematic illustration of OGOS chemical structure alteration during the thermal oxidation.

**Table 1:** Quantitative analysis of wide scan and deconvoluted high-resolution C1s XPS spectra of OGOS, OGOS-450 and NOG sample.

Sample	C/O	C %	(C=C) %	(C–C/C–H) %	(C–O) %	(C=O) %	(O–C=O) %
OGOS	2.27	69.44	51.22	6.02	38.03	7.03	3.14
OGOS-450	3.48	77.72	79.20	–	17	2.27	1.53
NOG	1.67	62.57	26.7	8.48	1.34	54.2	9.32

### Self-assembled NOG fibers

#### Formation mechanism

**Figure 4:**
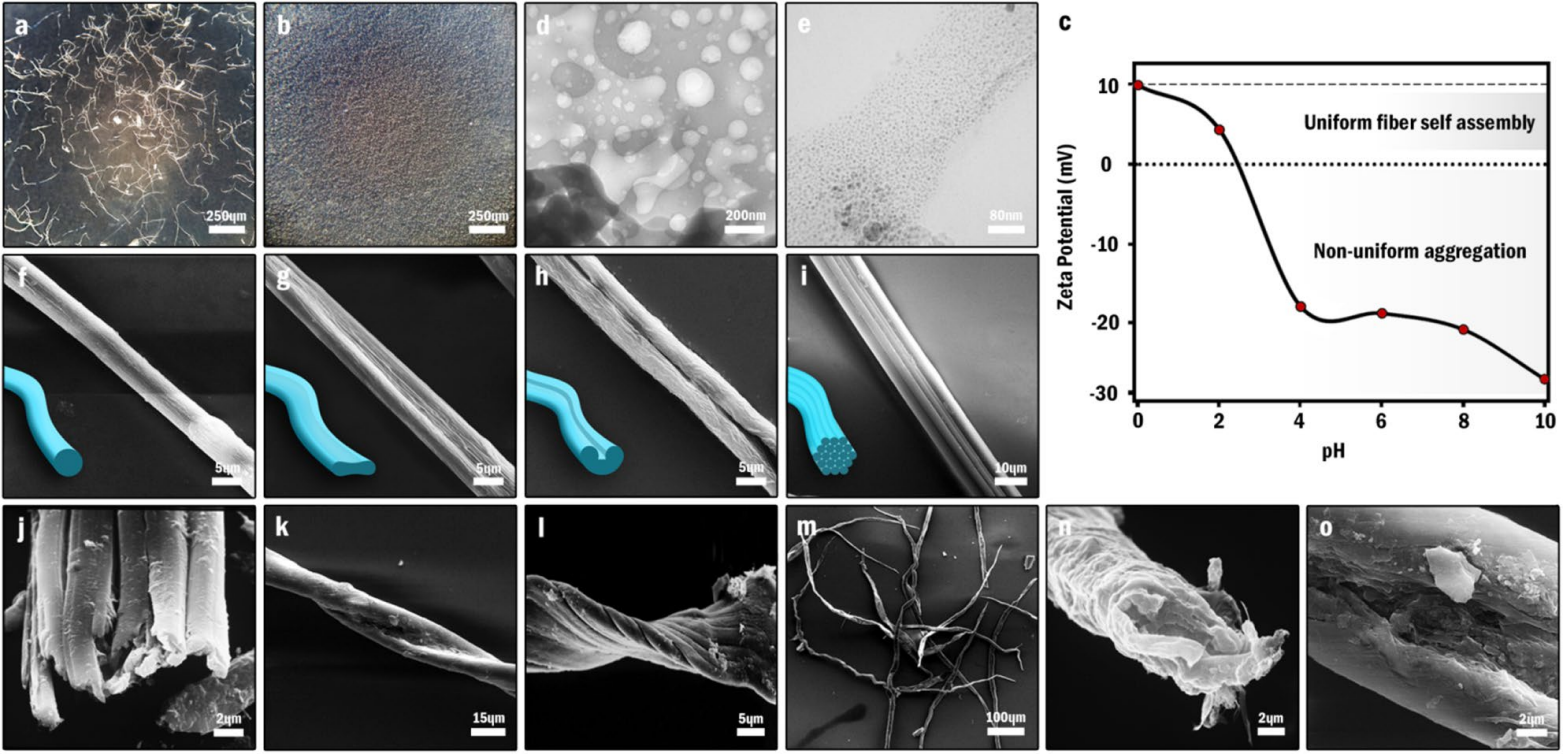
Transformation of NOG nanosheets into NOG fibers, (**a**–**b**) OM images of dried NOG fibers and unshaped flocculated particles on glass slide obtained from sonication of NOG nanosheets at pH: 0 and pH: 8 respectively. (**c**) Zeta potential of NOG dispersion (1 mg/mL) as a function of pH. (**d**–**e**) TEM images of the NOG nanosheets before sonication in acidic solution, and the resulting quantum dots after a 5 min-sonication in acidic solution (HCl 6 M) respectively. (**f**–**i**) SEM images of CF, CTF, KSF, and MSF respectively. (**j**) Cross-section SEM image of MSF (**k**–**l**) SEM images of twisted KSF and MSF. (**m**) A low magnification SEM image of NOG fibers (**n**) Cross-section SEM image of CTF (**o**) Extreme close-up SEM image of KSF indicating the porous structure of the central channel.

As previously stated, although the NOG nanosheets tended to form unshaped aggregations in pH > 2, the acidification of dispersion to pH < 2 triggered the formation of some flexible transparent fibers (Fig. 4a,b); hence, it was hypothesized that the rationale of this self-assembly falls into the hands of ionic interactions and the surface charges. In an attempt to find the mechanism of this self-assembly, the variations of zeta potential as a function of dispersion pH were investigated (Fig. 4c). In this study, the isoelectric point (pH_PZC_) of NOG nanosheets found to be 2.4; therefore, in the pH range of 0.0 to 3.1 which the ζ-potential lays in the range of 0 ± 10 mV, the dispersion must have the least stability and the nanosheets must be aggregated rapidly^43^. Actually, by lowering the pH, the negatively charged carboxylate moieties are protonated and the attractive forces including hydrogen-bondings between abundant oxygen-containing functional groups, π-π interactions of aromatic rings, and Van der Waals forces supersede the electrostatic repulsive forces. However, if the dominance of attractive forces was the primary cause of this self-assembly, the NOG fibers had to be produced in other pH values as well; seeing that, every dispersion exhibiting a ζ-potential up to ± 30 mV tend to form aggregation sooner or later^43^. Additionally, the dispersions with higher ζ-potentials are more prone to form such orderly fibers compared to those with lower ζ-potentials; because the dispersions possessing higher ζ values show longer aggregation time and accordingly, the nanosheets can be assembled more regularly. However, the NOG fibers, fabricated in dispersions within the range of 0 to + 10 mV, were more uniform than unshaped flocculated particles of other pH values. Given these points, it was understood that such attractions between the NOG nanosheets cannot be the mechanism of this transformation.

In fact, sonication of the positively charged NOG nanosheets below the pH of 2.4 found to be the principal driving force of this fabrication. In other words, during the sonication, the sound waves give rise to continual pressure variations and cavitation in a medium which grow and collapse repetitively and bring about extreme strain rate in a liquid^44^. In the case of carbon nanomaterials' dispersion, these persistent implosions trigger friction forces, stretch the carbon framework and lead to bond fracture^45^. Sonication-induced decomposition of carbon nanomaterials such as SWCNT and GO into smaller fragments and a series of hydrophilic polycyclic aromatic hydrocarbons (PAHs) like hydroxylated analogs of naphthalene, anthracene, and pyrene has been substantiated to be feasible in strong acid solutions^45^. Indeed, the acid cuts the carbon network into smaller fragments by the annihilation of damaging sites and subsequently hydrogenizes the dangling bonds and decomposes the fragments into PAHs. Accordingly, since NOG benefits from a highly defected structure that is laden with lots of oxygen functionalities, it is more vulnerable to sonication in the acidic medium compared to GO and SWCNT and thus, is fractured into small fragments and PAHs more conveniently. TEM analyses of two aliquots obtained from the synthesis pot before and after the sonication showed that NOG nanosheets are shattered into 1–4 nm quantum dots as a result of sonication in acidic medium (Fig. 4d,e).

Alternatively, it has been reported that these smaller fragments can be dehydrated and reconstituted into larger carbon structures by further sonication^45^. Therefore, it is speculated that the PAHs and tiny GO fragments produced from the fragmentation of some NOG nanosheets are reconstituted into larger carbon nanostructures like CNTs and act as seeds for assembly of other NOG fibers’ building blocks comprising PAHs, small-sized GO fragments and, other NOG nanosheets. Investigation of the effect of hydrochloric acid concentration on the formation of NOG fibers provides a better insight into the proposed mechanism (Fig. S2). Firstly, when NOG nanosheets were sonicated in HCl (0.01 M, pH 2), the self-assembly was not complete and a lot of unassembled particles were observed. Secondly, the increment of HCl concentration not only increased the efficiency of the self-assembly but also ameliorated the neatness of the fibers. In fact, at low concentrations, the presence of large unbroken NOG nanosheets in the fibers' composition engenders a rough surface. However, as the concentration increases, NOG nanosheets are broken into smaller pieces and the resulting fibers become smoother and shinier.

NOG fibers also exhibited great chemical stability and were neither disassembled nor decomposed in different media including organic solvents with different polarities (Hexane, Toluene, Chloroform, Acetone, DMSO, Acetonitrile, and Methanol), bases (sodium hydroxide (1 M) and ammonia (30% w/w)), acidic and oxidizing solutions (hydrochloric acid (37% w/w), and hydrogen peroxide (30% w/w). These observations confirmed that the bondings, playing the major role in the stability of fibers are chemical and cannot be dissociated readily.

#### Morphology

With the purpose of shedding a light upon the structure and morphology of NOG fibers, FE-SEM imaging was exploited. First, FE-SEM micrographs of individual fibers revealed their polymorphic nature with four distinctive morphological structures (Fig. 4f–i). Indeed, such structures have been reported since years ago for natural and synthetic fibers such as cotton and nylon and regarded as one of their identifying characteristics^46,47^. As a rule, these structures are classified based on the appearance and cross-section's shape of the fibers. In a similar fashion, as far as NOG fibers are concerned, they were classified into single-strand and multi-strand fibers (MSFs). On the one hand, the single-strand group consisted of circular fibers (CFs), collapsed tube fibers (CTFs), and kidney-shaped fibers (KSFs); on the other hand, multi-strand group entailed fibers composed of a bunch of rod-shaped fibers clung to each other from the flanks (Fig. 4j). Moreover, the number of constitutive strands of multi-strand fibers varied from one to another. In terms of structural distribution, collapsed tube fibers dominated the majority of the population and kidney-shaped fibers, multi-strand fibers, and circular fibers were in the next places respectively. Determinant dimensions of each structure were measured as well. Note, the following reported intervals indicate the size distribution of each case and should not be mistaken with the uncertainties of the measurements. On this subject, CTFs, as the most plentiful constituents of the samples, possessed the width of 13.1 ± 1.2 µm and thickness of 4.0 ± 0.5 µm. Furthermore, the CFs had the diameter of 8.9 ± 1.0 µm and MSFs were 14.1 ± 2.2 µm wide and their constitutive fibers had the diameter of 4.6 ± 0.6 µm. Talking of KSF, it was revealed that the lateral lobs have the identical width of 5.7 ± 2.1 µm and thus are symmetrical; besides, the values of vertical thickness, horizontal thickness and central channel width were determined 8.6 ± 1.1 µm, 16.0 ± 5.2 µm and 4.3 ± 1.5 µm respectively. The width's sequence of the fibers was also revealed as KSF > MSF > CTF > CF. The length distribution of the fibers was also investigated regardless of their structures and was found 0.9 ± 0.1 mm. The macroscopic twists, so-called convolutions, tended to occur along all types of fibers either single-strand or multi-strand; however, owing to the symmetrical structure of CFs, the occurrence of convolutions could not be detected (Fig. 4k,l). Although the flexibility of the fibers could be observed even with the naked eyes, the twisting and bending of the fibers in microscopy images witnessed their flexibility as well (Fig. 4m). The cross-section of the collapsed tube fiber disclosed its multilayer structure and proved the presence of the NOG nanosheets in the structure of the fibers (Fig. 4n). The channel existing in the middle of the kidney-shaped fiber also showed a great porosity which coupled with the nanoporous structure of NOG nanosheets provides a high specific surface area (Fig. 4o).

#### Body colorations

**Figure 5:**
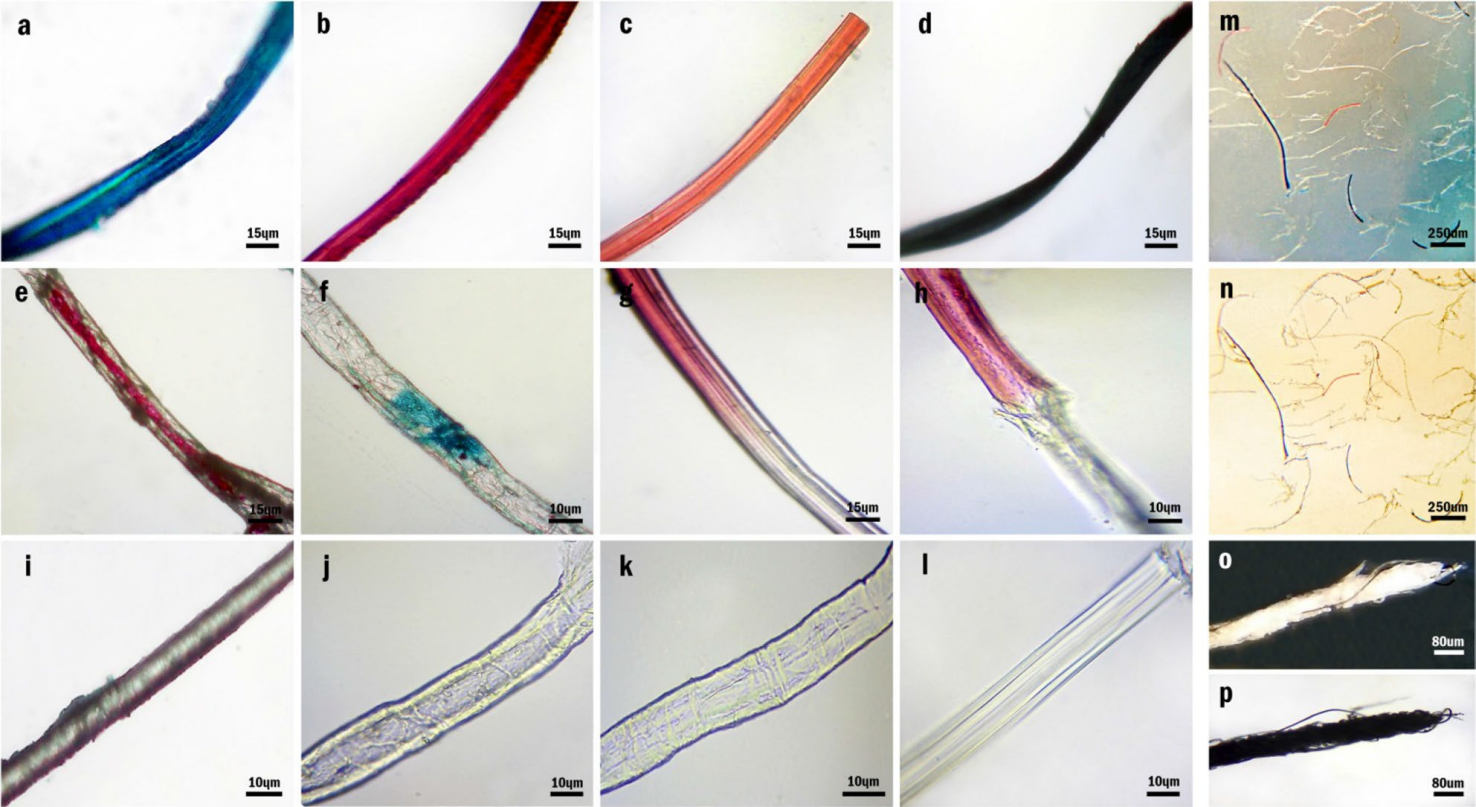
Body coloration of NOG fibers, (**a–d**) OM images of single-colored blue, dark red, light red, dark green fibers. (**e–h**) OM images of dual-colored fibers (**i–l**) OM images of colorless CF, KSF, CTF, and MSF (**m–n**) OM images of NOG fibers under episcopic and diascopic illuminations. (**o–p**) OM images of self-assembled yarn under episcopic and diascopic illuminations.

Optical microscopy (OM) imaging was carried out in order to analyze the appearance of the fibers. To begin with, all of the fibers, regardless of their colors and structures, were glossy and transmitted the light perfectly. With respect to body coloration, the fibers were generally classified into three groups of single-colored (Fig. 5a–d), dual-colored (Fig. 5e–h) and colorless (Fig. 5i–l) fibers. Single-colored fibers encompassed one of the four colors of dark blue, dark red, light red, and dark green. Besides, although, the dark green fibers appeared to be black at low magnifications, their green color was detected by closer observations. (Fig. S3) On the other hand, the dual-colored category entailed fibers whose colorations were a combination of colorless regions with one of the foregoing colors. Regarding the fibers distribution in terms of body coloration, colorless fibers constituted the majority of the fibers; however, the single-colored and dual-colored fibers were in minority. Furthermore, the differences between the appearances of four fiber structures were significant enough to distinguish one from another using OM (Fig. 5i–l). Besides, the fibers coloration remained the same by changing the illumination mode from diascopic to episcopic (Fig. 5m,n). This shows that the mechanism of light interaction with the fibers is not pertinent to this matter that whether it is reflected from or transmitted through the fibers and thus, the coloration mechanism is the same in both illumination modes. Having said that, a self-assembled yarn, made of many randomly twisted colorless NOG fibers, was also beheld; although it looked white and shiny under episcopic illumination, it was totally black under diascopic illumination (Fig. 5o,p). In fact, even though the colorless fibers are transparent, the intensity of the transmitted light from each fiber is decreased on account of partial reflection; hence, after multiple successive transmissions and reflections, the intensity of the light dwindles significantly and the yarn is observed black.

#### Marginal colorations

**Figure 6:**
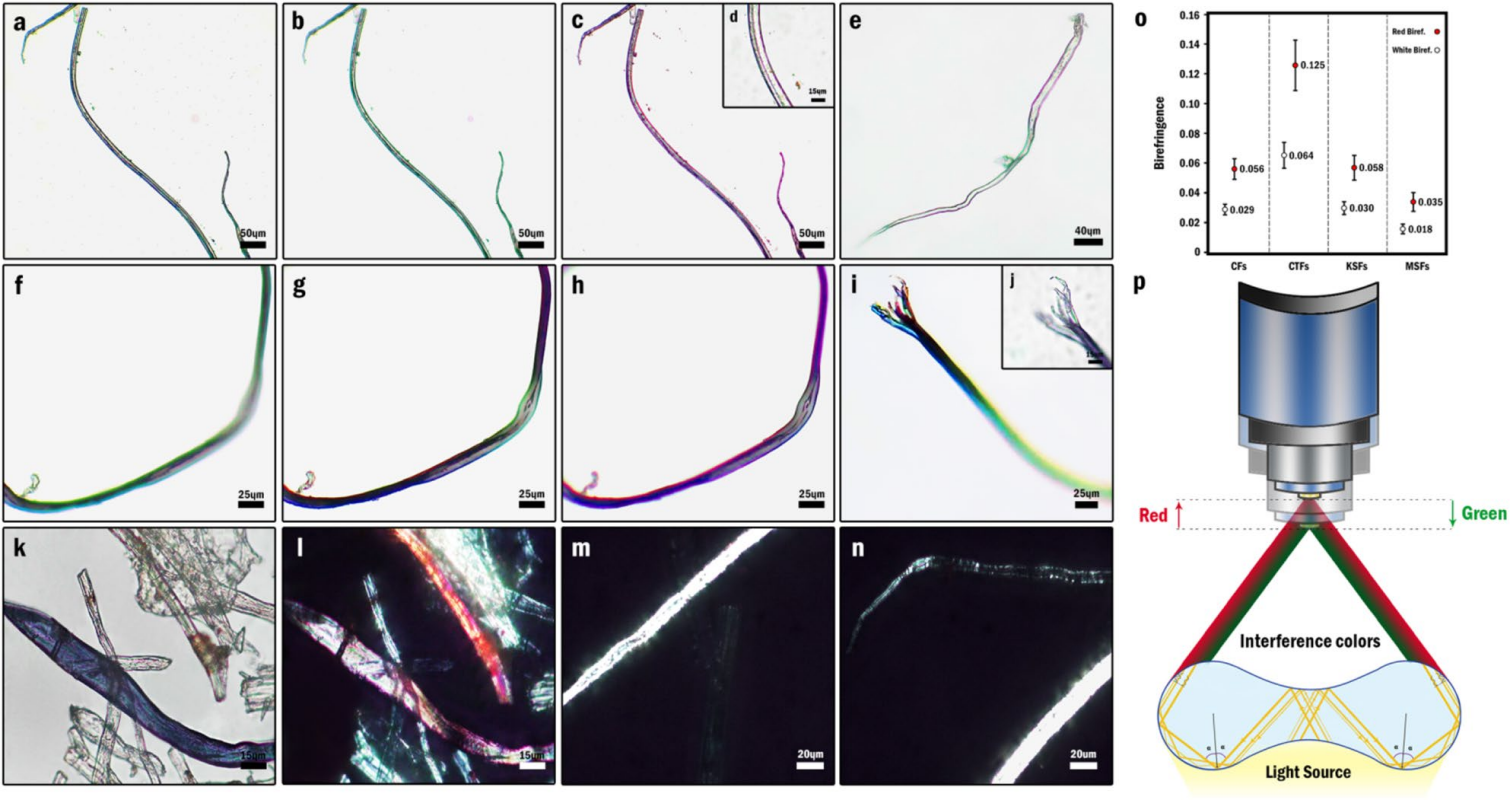
Marginal coloration and birefringence of dried NOG fibers, (**a**) OM image of colorless MSF in fine focus (**b**) OM image of colorless MSF with green marginal color after a trivial increase of working distance from the fine focus. (**c**) OM image of colorless MSF in fine focus with red marginal color after trivial reduction of working distance from the fine focus. (**d**) The red marginal coloration of colorless MSF in higher magnification. (**e**) Red and green marginal coloration in a colorless CTF which is not located in a single plane. (**f**–**h**) alteration of iridescent marginal coloration by reduction of working distance in a dark green KSF which is not located in a single plane. (**i**) Multicolored marginal coloration of MSF terminal (**j**) Absence of multicolored marginal coloration of dark green MSF terminal in higher magnification (**k**–**l**) OM and POM images of NOG fibers indicating strong red and white birefringence (**m**–**n**) POM images of KSF and MSF after 45◦ rotation of the specimen between the cross polarizers (**o**) Diagram indicating the birefringence intervals of different fibers' structures (**p**) Schematic illustration of the total internal reflection and light interference at concave interfaces of CTF.

The OM studies of the fibers were carried out both in wet and dry media. In contrast to the wet samples, a conspicuous narrow line of iridescent colors appeared on the marginal regions of the fibers in dried samples. As an illustration, when the whole body of a colorless fiber was attached to the slide's surface and placed in a single focal plane, a partial array of blue, green, and red colors was observed on borders after adjusting of resolution to the sharpest focus (Fig. 6a). However, by the slight movement of the objective lenses toward and away from the specimen using the fine adjustment knob, the marginal color-array was transformed into red and green respectively (Fig. 6b,c,d). This revealed that the wavelength of the observed light depends on the working distance (the distance between the objective lens and the specimen) and can be changed easily in a wide range of colors by precise adjustment of the spacing. Besides, the single marginal color was merely achievable providing that the whole body of the fiber was placed in a single plane. With this in mind, the multi-colored appearance of the margins which were beheld with the fixed objective lens emanated from the fact that different parts of the fiber were placed in different heights and were not attached utterly to the surface of the slide (Fig. 6e–h); therefore, it could be feasible to fix the fiber in an intended position in order to observe the desired coloration. This type of coloration was seen in all fibers with different colors but was extremely conspicuous in colorless fibers owing to its considerable contrast with the body color. Furthermore, since the MSF is consisted of smaller fibers, by the placement of this fiber's terminal in the space, each of the constitutive fibers occupied a particular height and exhibited different marginal colorations beside each other (Fig. 6i). Not to mention, such marginal colorations could be only beheld clearly in a particular height from the specimen and since the working distance decreases as the magnification increases, observation of this coloration in a fine focus was not possible in all magnifications (Fig. 6j).

The justification of this marginal coloration is most likely rooted in light interference at micro-scale concave interfaces caused by total internal reflection (TIR)^48^. Actually, when light reaches an interface with a less dense medium (lower refractive index), it is entirely reflected provided that its incidence angle exceeds the critical angle. In this regard, the light is reflected numerously from different interfaces inside of the dense medium until it reaches the critical angle and passes the interface entirely. As a result of such reflections at a concave optical interface, different light beams in different trajectories can interfere to generate various patterns of colors. This mechanism has been suggested for the creation of structural coloration within a wide range of materials comprising sessile droplets, biphasic droplets, solid particles, and polymeric microstructures with both curved and flat sides. Given these points, when NOG fibers are in the air which has the lowest refractive index (n = 1.000), TIR could occur inside of the fibers' body; consequently, the light beams, traveling in different trajectories, are reflected from the concave interfaces of the lateral parts, interfere with each other and cause the aforementioned iridescent marginal coloration (Fig. 6p). However, on account of the higher refractive index of water compared to the air, TIR could not happen inside the fibers' body in a wet medium and therefore, no marginal coloration can be seen.

#### Birefringence

Strong birefringence of the fibers indicated their highly anisotropic structure and internal orientational order. Besides, despite the fact that the majority of the colorless fibers showed strong white birefringence, some fibers exhibited the interference color of red (Fig. 6k–n). The birefringence of the colorless fibers with different structures was estimated by using Michel Levy color chart and the average thicknesses obtained from SEM imaging (Fig. 6o). However, since the thickness of the fibers varied from one to another, the birefringence of each structure was reported as an interval instead of a constant particular value.

#### Photoluminescence

Strong fluorescence throughout the UV–visible and near IR regions was discovered to be another interesting attribute of NOG fibers (Fig. S4a). As a matter of fact, since the emission spectra of the NOG nanosheets and the fibers bore a close resemblance to each other, it seems that the fibers have inherited this property from the NOG nanosheets (Fig. S5a). This analogy also reflects that the sizes of the *sp*^2^ clusters liable for the diverse fluorescence emissions of the NOG nanosheets do not change significantly throughout the fragmentation and self-assembly steps. Besides, the similarities of the NOG nanosheets and the fibers spectra can also be interpreted on behalf of the placement of the NOG nanosheets in the fibers' constitution which was proved by FE-SEM images in previous sections. Alternatively, the emission spectra of NOG fibers and nanosheets with several consecutive ʎ_ex_s in the region of 300–800 nm disclosed that the fibers exhibit UL together with a few other fluorescence emissions as well (Fig. S4b–S4l, S5b–S5l).

**Figure 7:**
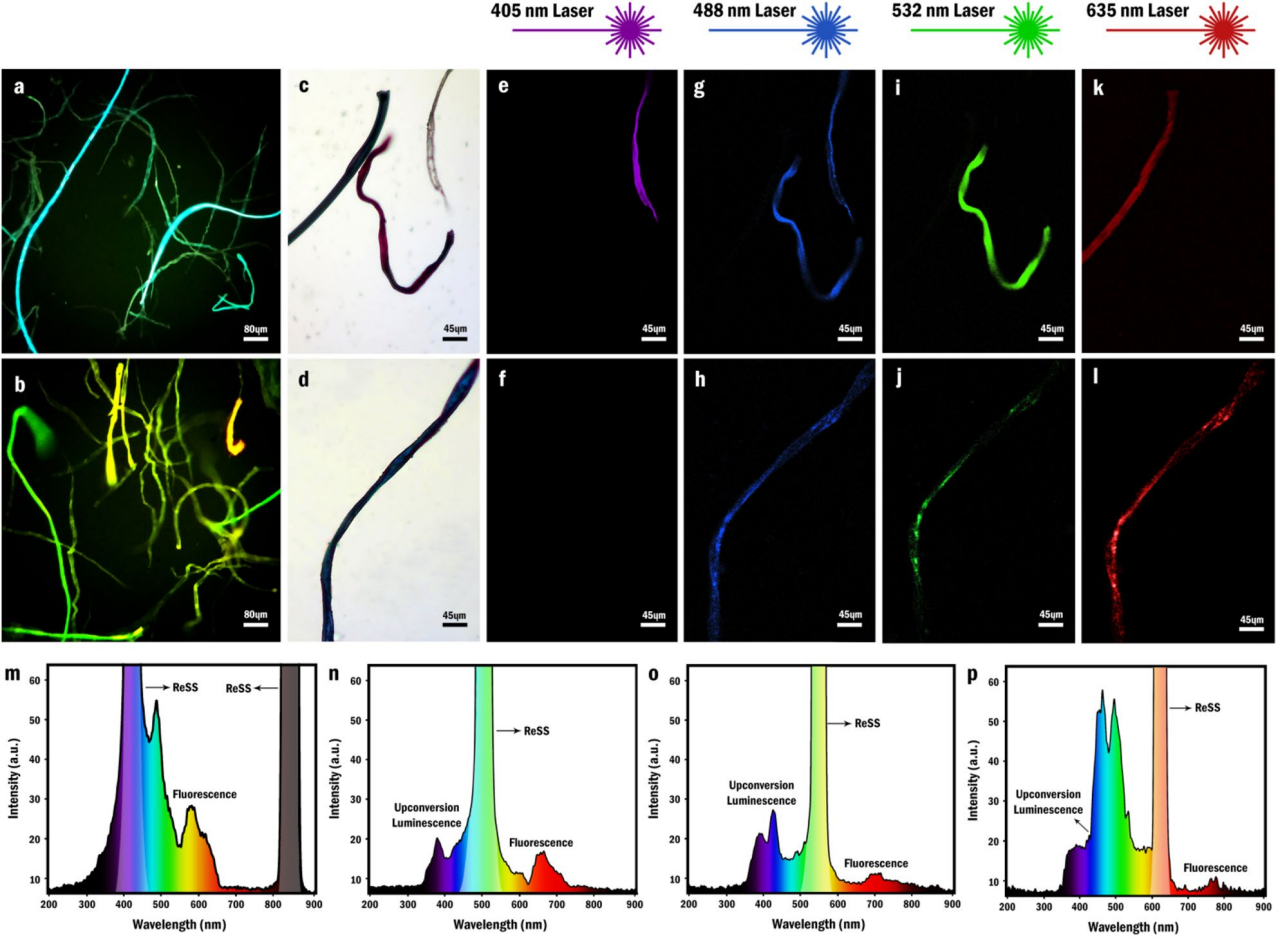
Fluorescence microscopy and confocal laser scanning microscopy of dried NOG fibers, (**a**) FLM image of NOG fibers' cyan blue fluorescence by using UV filter (**b**) FLM image of NOG fibers' green, yellow and red emissions by using blue filter (**c**–**d**) OM images of colorless, blue, red and dark green fibers (**e**–**f**) Violet channel CLSM images of NOG fibers (**g**–**h**) Blue channel CLSM images of NOG fibers (**i**–**j**) Green channel CLSM images of NOG fibers (**k-l**) Red channel CLSM images of NOG fibers (**m**–**p**) PL emission spectra of the NOG fibers with ʎ_ex_ of 405, 488, 532 and 635 nm respectively.

In an effort to visualize the PL of the NOG fibers and with the purpose of scrutinizing the behavior of different fibers’ colorations in this phenomenon, Epifluorescence Microscopy (EFM) and Confocal Laser Scanning Microscopy (CLSM) were utilized. In this regard, although all of the fibers’ PL looked brilliant cyan-blue with UV filter, the observations with blue filter revealed that fibers emit green, yellow, and red lights as well, which are in compliance with the aforementioned emission spectra (Fig. 7a,b). Alternatively, CLSM with 405, 488, 532, and 635 nm lasers as the excitation sources reflected the presence of different fluorophores in the structure of NOG fibers. To clarify, first of all, the 405 nm laser (violet light) selectively excited the colorless fibers among other fibers with different colors (Fig. 7c–f) (Fig. S6a, S6b). Secondly, all of the fibers with different colors tended to emit light under illumination by 488 nm laser (blue light) except green fibers (Fig. 7g,h) (Fig. S6c). Besides, it was revealed that 532 nm laser (green light) is an efficient light source for selective excitation of the red regions both in single-colored and dual-colored fibers (Fig. 7i) (Fig. S6d). Alternatively, green and blue fibers were the only ones that absorbed the red light from 635 nm laser to exhibit PL (Fig. 7k,l) (Fig. S6e). The simultaneous emission of the colorless, red and blue fibers under illumination by 488 nm laser, together with the PL of the blue fibers after excitation by each of the blue, green and red lasers disclosed that the NOG fibers can contain different components in their structures and are not necessarily composed of a single ingredient (Fig. 7g,h,j,l). The fibers' emission spectra, corresponding to the ʎ_ex_ of each lasers, were provided as well (Fig. 7m–p). Given these points, it seems reasonable to deduce that the selective excitation of the fibers originates from the existence of different specific components with different colors which not only are responsible for PL of the fibers but also play the crucial role in their coloration.

#### The origin of the body colorations and UL

Among various physical phenomena which have been taken into consideration as the origin of the materials’ coloration, one of the two phenomena of spectrally selective light absorption by chromophores and light interference in periodic structures such as GO photonic crystals^25^ are most likely the underlying mechanism of the NOG fibers' coloration. As previously stated, the correlation between the wavelengths of the absorbed lights and the observed colors in the fibers was conspicuous, which reflects that the fibers' different colorations come from the spectrally selective light absorption by different chromophores existed in the structure of the fibers. Tending to substantiate the aforementioned assertion, the excitation spectra of the fibers for different consecutive emission wavelengths were investigated. Actually, since the sensitivity of the PL spectroscopy is much higher than the UV/visible absorption spectroscopy, the detection of the photoluminescent species with very low concentrations is carried out more precisely with PL spectroscopy. Moreover, since the excitation spectra mostly resemble the absorption spectra, they were examined instead in order to detect the signals of the colorful species responsible for the coloration of the NOG fibers. In this way, the excitation spectra indicated that the fibers are constituted of at least six particular colorful components of yellow, orange, red, indigo, blue and green, which together with the colorless component result in diverse coloration and PL of the fibers. More explicitly, the UL and fluorescence peaks centered at ~ 500 nm (~ 2.48 eV), ~ 550 nm (~ 2.26 eV), ~ 600 nm (~ 2.07 eV), and ~ 650 nm were assigned to excitation of red, indigo, blue and green components and the fluorescence peaks at ~ 430 nm (~ 2.89 eV), ~ 390 nm (~ 3.19 eV) and ~ 320 nm (~ 3.89 eV) were attributed to excitation of orange, yellow and colorless components respectively (Fig. S7). The signals of all colorful components were also detected in NOG nanosheets excitation spectra, which indicate that these species are available in NOG nanosheets as well (Fig. S8). Besides, it can be considered as another witness on the placement of the NOG nanosheets in the composition of the fibers. The aforementioned observations about the presence of the colorful species are most likely attributed to the O-doped PAHs with different sizes of π-conjugated systems formed as a result of NOG nanosheets decomposition. In fact, tailoring of the PAHs' electronic structure with the purpose of acquiring novel optical properties comprising color and PL by means of bottom-up synthesis of oxygen-embedded π-conjugated systems has been well documented^49–51^. In this respect, a study focused on the O-annulation of PAHs for the synthesis of different compounds with diverse band gaps, colors and PL emissions has great conformity with the observations regarding the NOG fibers^52^. Therefore, it is speculated that since the formation of PAHs through ultrasonication of the graphenic nanosheets was reported to be feasible, the formation of their oxidized colorful derivatives from the fragmentation of the NOG nanosheets is plausible as well.

This explanation can also justify the UL of NOG nanosheets and fibers. To clarify, although several justifications comprising multiphoton active process^53^, anti-Stokes PL^54^, as well as phonon-assisted UL^55^ have been proposed for UL mechanism of carbon-based nanoparticles, pursuant to the aforesaid PL spectra, it is postulated that due to the intrinsic capability of the PAHs' derivatives as efficient sensitizers and annihilators in Triplet–Triplet Annihilation (TTA) system, they most likely account for the UL of NOG nanosheets and fibers^56^. In other words, at first, the colorful components exhibiting UL excitation signals including red, indigo, blue and green species operate as the sensitizers and absorb the low energy photons to populate their first excited triplet states through the intersystem crossing. Then, the species that merely exhibit fluorescence, including colorless, yellow, and orange components, function as the annihilators and after triplet–triplet annihilation give rise to UL photons. However, finding the exact mechanism quite involved time-resolved fluorescence spectroscopy which is outside the scope of the present investigation.

## Conclusion

All in all, a series of flexible self-assembled fibers with remarkable optical properties including high optical transparency, immutable body coloration, tunable interference marginal coloration, strong birefringence along with the fluorescence and upconversion emissions was synthesized through the sonication of thermally oxidized graphene oxide nanosheets in acidic medium. These free-standing polymorphic fibers were classified into four morphological structures of circular, collapsed-tube, and kidney-shaped and multi-strand fibers. They also exhibited extraordinary stability in strong basic, acidic and oxidizing media which designates them as a potential candidate for the development of chemosensors for corrosive environments. These outstanding features not only introduce NOG fiber as a versatile material to many promising multifunctional applications but also underlie several groundbreaking systematic investigations in the future.

## Materials and methods

### Materials

Graphite powder (particle diameter: 45 µm) and potassium permanganate (KMnO_4_; 99.0%) were supplied by Sigma-Aldrich Co. and sulfuric acid (H_2_SO_4_; 98%), phosphoric acid (H_3_PO_4_; 85%), hydrochloric acid (HCl; 37% in water) and hydrogen peroxide (H_2_O_2_; 30% in water) were purchased from Merck-Millipore Co. and used as received. PTFE membrane filter (pore diameter: 0.2 µm) was procured from Sartorius Stedim Co. and used as received.

### Instrumentation

Transmission electron microscopy (TEM) analysis was conducted by a Philips CM300 microscope with an acceleration voltage of 200 kV. X-ray photoelectron spectroscopy (XPS) was performed using a Thermo Scientific K-Alpha spectrometer equipped with a hemispherical analyzer and an Al K_α_ X-ray source (hʋ = 1486.6 eV) working in a vacuum better than 10^–7^ Pa. The XPS peaks were deconvoluted using Gaussian components after a Shirley background subtraction. The structure, size, and morphology of samples were studied by using a Tescan-Mira III field emission scanning electron microscope (FE-SEM) after sputter-coating of each sample with a ~ 7 nm gold layer. The UV/Vis absorption and photoluminescence spectra were recorded by an Agilent 8453 UV–Visible spectrophotometer and a Perkin Elmer, LS50B luminescence spectrometer, respectively. A Leica TCS SP5 confocal laser scanning microscope together with a Bell FLUO3 fluorescence microscope was used for exploring the PL of NOG fibers. The zeta potential measurements of NOG samples were carried out by a Malvern Zetasizer Nano ZS dynamic light scattering spectrophotometer. Different samples of NOG fibers were viewed under a Nikon ECLIPSE LV100N POL polarized optical microscope and a Zeiss Axiostar Plus bright field microscope in order to examine the fibers coloration, structure, and birefringence. A Q600 TA Instrument thermal gravimetric analyzer was utilized for following the mass alterations of OGOS samples by annealing in pure argon and air atmospheres. Thermal treatment of all samples was carried out by a Nabertherm L 1/12 air muffle furnace.

### Synthesis of graphene oxide nanosheets

GO nanosheets were synthesized based on the improved Hummers' method^57^, albeit with some modifications. To this end, 3.0 g of graphite powder was added into 400 ml of 4:1 mixture of H_2_SO_4_/H_3_PO_4_ at room temperature and the mixture was placed in an ice bath and cooled down to 0 °C. Then, 18 g of KMnO_4_ was gradually added into the mixture maintaining the temperature below 10 °C. The reaction pot was then placed in an oil bath and stirred for 12 h at 50 °C. In the next step, the reaction mixture was cooled down to room temperature and poured onto ~ 400 ml of ice. In order to quench the reaction, 3.0 ml of H_2_O_2_ was gently added under stirring. In order to ensure the utter exfoliation of the graphite oxide, the resulting bright yellow suspension was sonicated for one hour. Then, the mixture was centrifuged (4000 rpm for 15 min), the supernatant decanted away and the remaining solid was washed three times with HCl (1 M) and once with deionized water respectively; each time, the mixture was centrifuged (4000 rpm for 15 min) and the supernatant decanted away. Eventually, a pasty light brown product was obtained and dried under vacuum for two days at room temperature.

### Preparation of orderly graphene oxide sediment (OGOS)

First, 100 mg of GO powder was sonicated in deionized water to form a stable and clear (1 mg/mL) dispersion. Afterward, so as to incorporate the GO nanosheets into lamellar structures with high alignment, the dispersion was centrifuged (12,000 rpm for 15 min) and the supernatant was decanted away and the whole pasty precipitate was redispersed in 50 mL of deionized water by vigorous shaking. The next step resembles the crystallization process and the duration of drying determines the stacking order of GO sheets in OGOS. Subsequently, the resulting viscous dispersion was poured into a petri dish (diameter, 10 cm) and gently dried in a vacuum desiccator for two days in order to form a uniform thin film of GO (OGOS) with the thickness of ~ 10 µm that was detached and used for the next step.

### Synthesis of nanoporous over-oxidized graphene (NOG)

First, 100 mg of OGOS was placed in a round porcelain crucible (top O.D., 30 mm), its aperture was closed with a suitable porcelain cap and then it was heated in an air muffle furnace up to 800 °C in 40 min (20 °C/min). The sample was then taken out from the furnace immediately after it reached 800 °C and subsequently, was cooled down to room temperature by airflow. In this way, ~ 6 mg of pale yellow brittle flakes were obtained as the final product.

### Synthesis of NOG fibers

To begin with, 2 mL of HCl solution (6.0 M) was added to 6 mg of NOG powder in a test tube and then it was sonicated in an ultrasonic bath for 2 min; afterward, it was taken out from the bath and stirred gently for 2 min. The two latter steps were repeated 8 times successively in order to obtain the highest amount of NOG fibers. Eventually, the yielded fibers were separated from the solution by vacuum filtration through a PTFE membrane filter (Pore size: 0.25 µm) and washed with 200 µL of deionized water. With the purpose of drying, at first, the fibers were transferred from the membrane onto the surface of a glass slide by suspending in 100 µL of deionized water and then were dried under vacuum for 1 h at room temperature.

## Supplementary Information

Supplementary Information
