# Supplementary material for: Self-assembled graphene-based microfibers with eclectic optical properties
Source: Sci Rep. 2021 Mar 9;11:5451. doi: 10.1038/s41598-021-84940-0 (PMC7943562; doi:10.1038/s41598-021-84940-0)
Supplement: Supplementary file 1 — Supplementary Information [file 41598_2021_84940_MOESM1_ESM.pdf]

Supplementary Information

Self-Assembled Graphene-Based Microfibers with Eclectic Optical Properties

Mahdi Ghamsari, Tayyebeh Madrakian, Mazaher Ahmadi, Abbas Afkhami*

Faculty of Chemistry, Bu-Ali Sina University, Hamedan, 6517838695, Tel/Fax:+98-8138380709, IRAN

Table of Contents

Figure S1. TGA curves of two OGOS samples heated in air and N_2 atmospheres..............................S2

Figure S2. Effect of HCl concentration on the self-assembly of NOG nanosheets..............................S3

Figure S3. OM images of dark green and red NOG fibers with low and high magnifications ………S4

Figure S4. Emission spectra of NOG fibers in neutral aqueous medium………………….…………S5

Figure S5. Emission spectra of NOG nanosheets in neutral aqueous medium ………..…………….S6

Figure S6. OM and CLSM images of red dual color NOG fiber……………………………………..S7

Figure S7. Excitation spectra of NOG fibers in neutral aqueous medium..…...……………………...S8

Figure S8. Excitation spectra of NOG nanosheets in neutral aqueous medium….…………………...S9

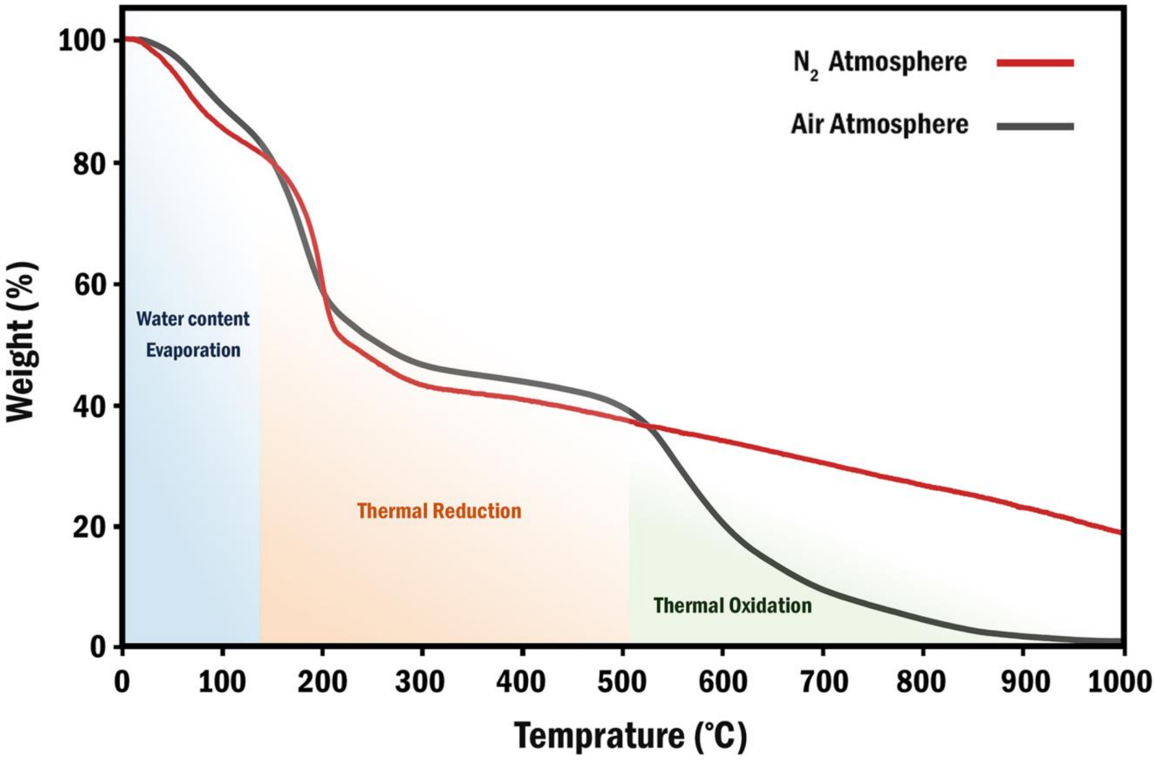

Figure S1. TGA curves of two OGOS samples heated in air and N_2 atmospheres.

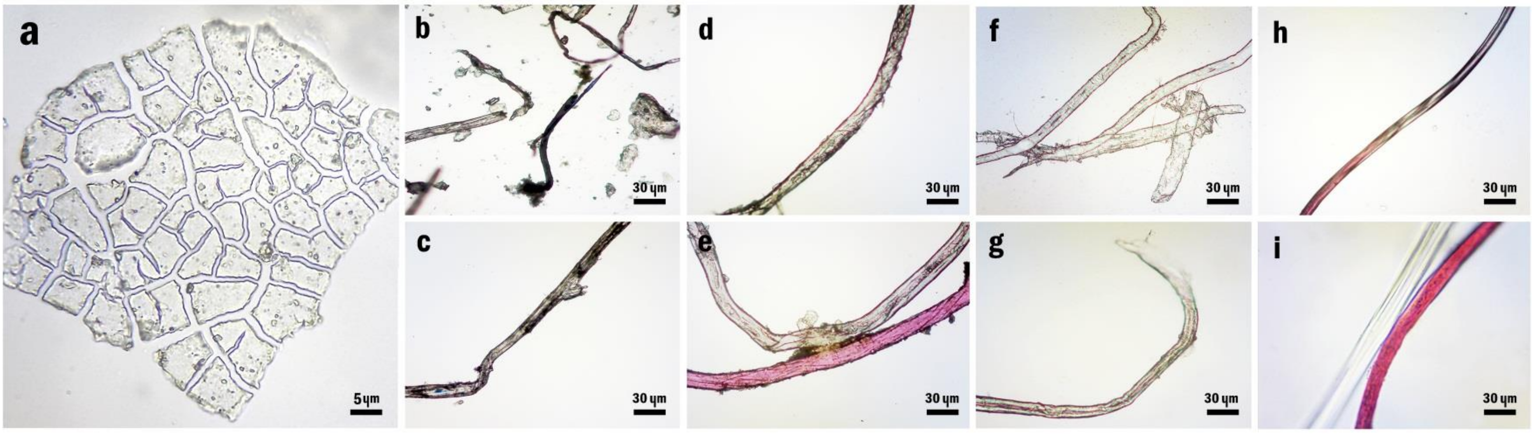

Figure S2. Effect of HCl concentration on the self-assembly of NOG nanosheets.

(a) Unsonicated NOG nanosheets obtained from HCl (0.01 M) dispersion. **(b-i)** Final products of the self-assembly process using HCl (0.01 M), (0.1 M), (1 M), (2M), (3 M), (4 M), (5 M), and (6 M) respectively.

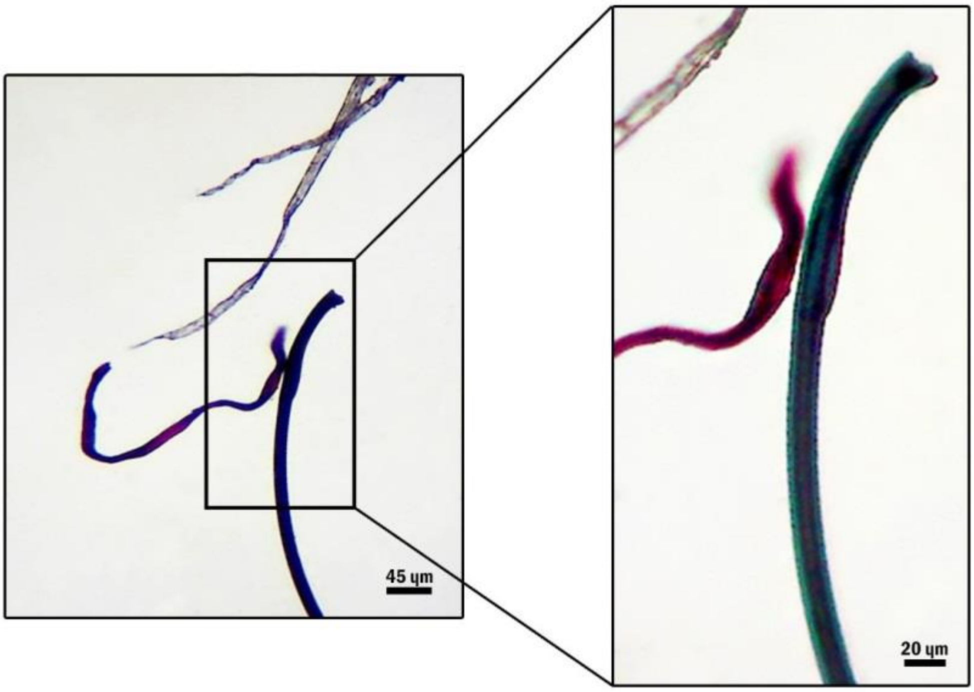

Figure S3. OM images of dark green and red NOG fibers with low and high magnifications.

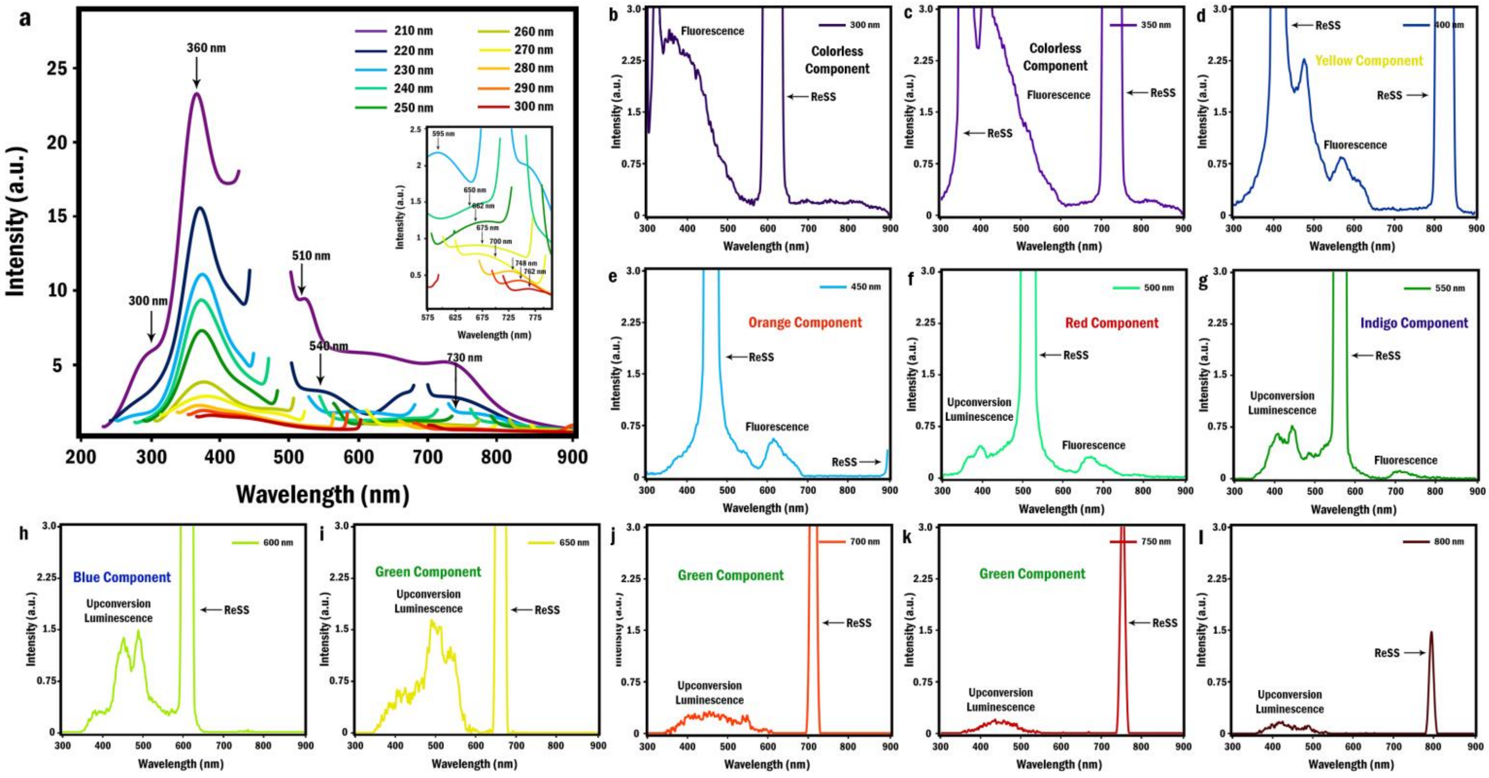

Figure S4. (a) Emission spectra of NOG fibers in neutral aqueous medium with different consecutive UV excitation wavelengths. Raman and Reighley scattering signals were eliminated for clarity. **(b-l)** Emission spectra of NOG nanosheets in neutral aqueous medium for several consecutive excitation wavelengths in 300-800 nm interval.

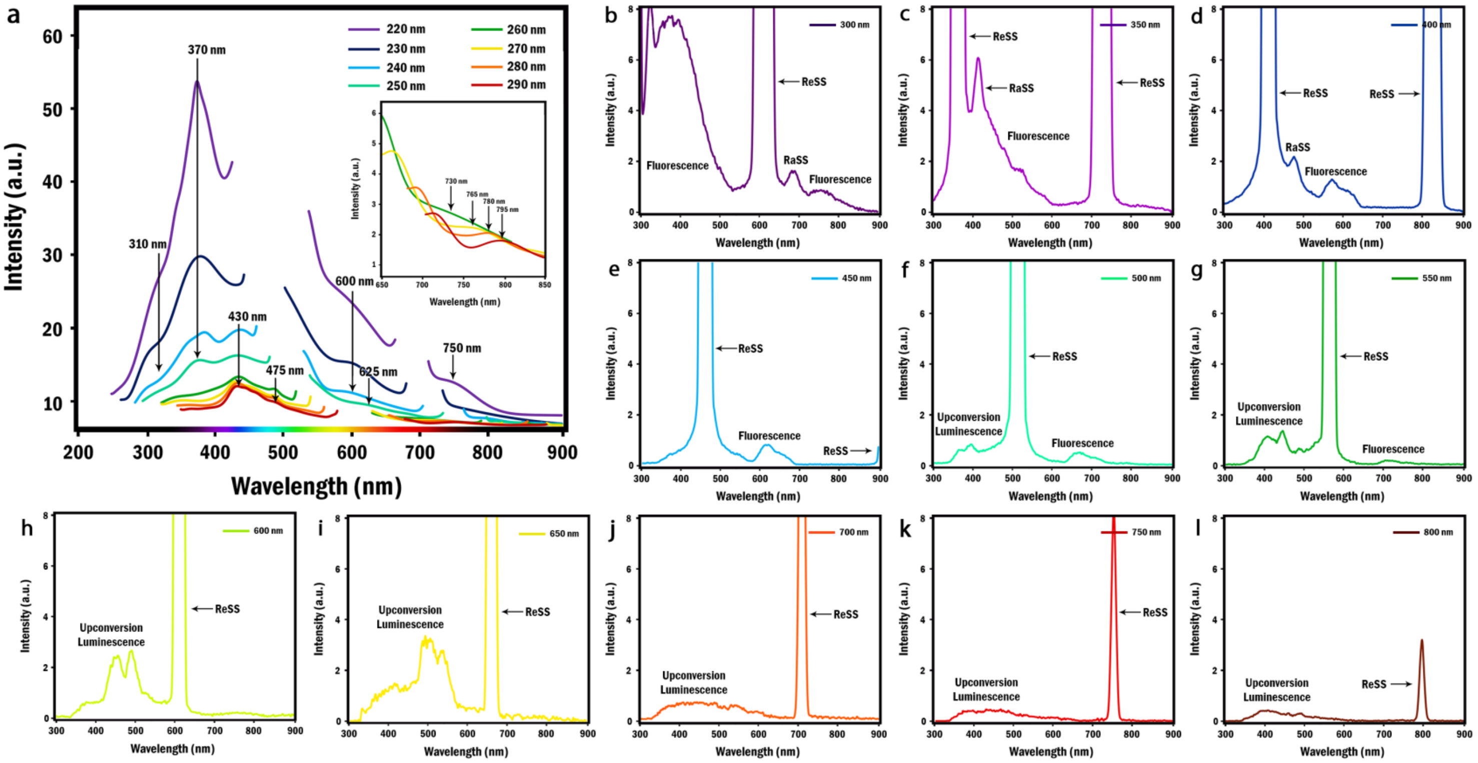

Figure S5. (a) Emission spectra of NOG nanosheets in neutral aqueous medium with different consecutive UV excitation wavelengths. Raman and Reighley scattering signals were eliminated for clarity. **(b-l)** Emission spectra of NOG nanosheets in neutral aqueous medium for several consecutive excitation wavelengths in 300-800 nm interval.

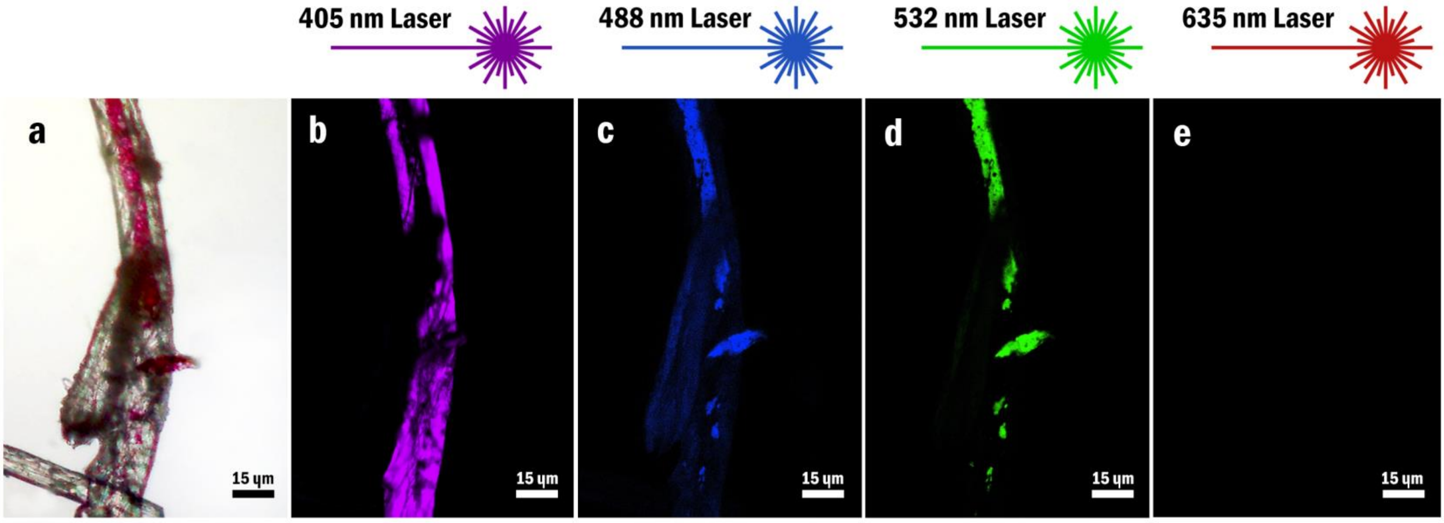

Figure S6. a) OM image of red dual color NOG fiber **(b-e)** CLSM image of red dual color NOG fiber with violet, blue, green and red channels respectively.

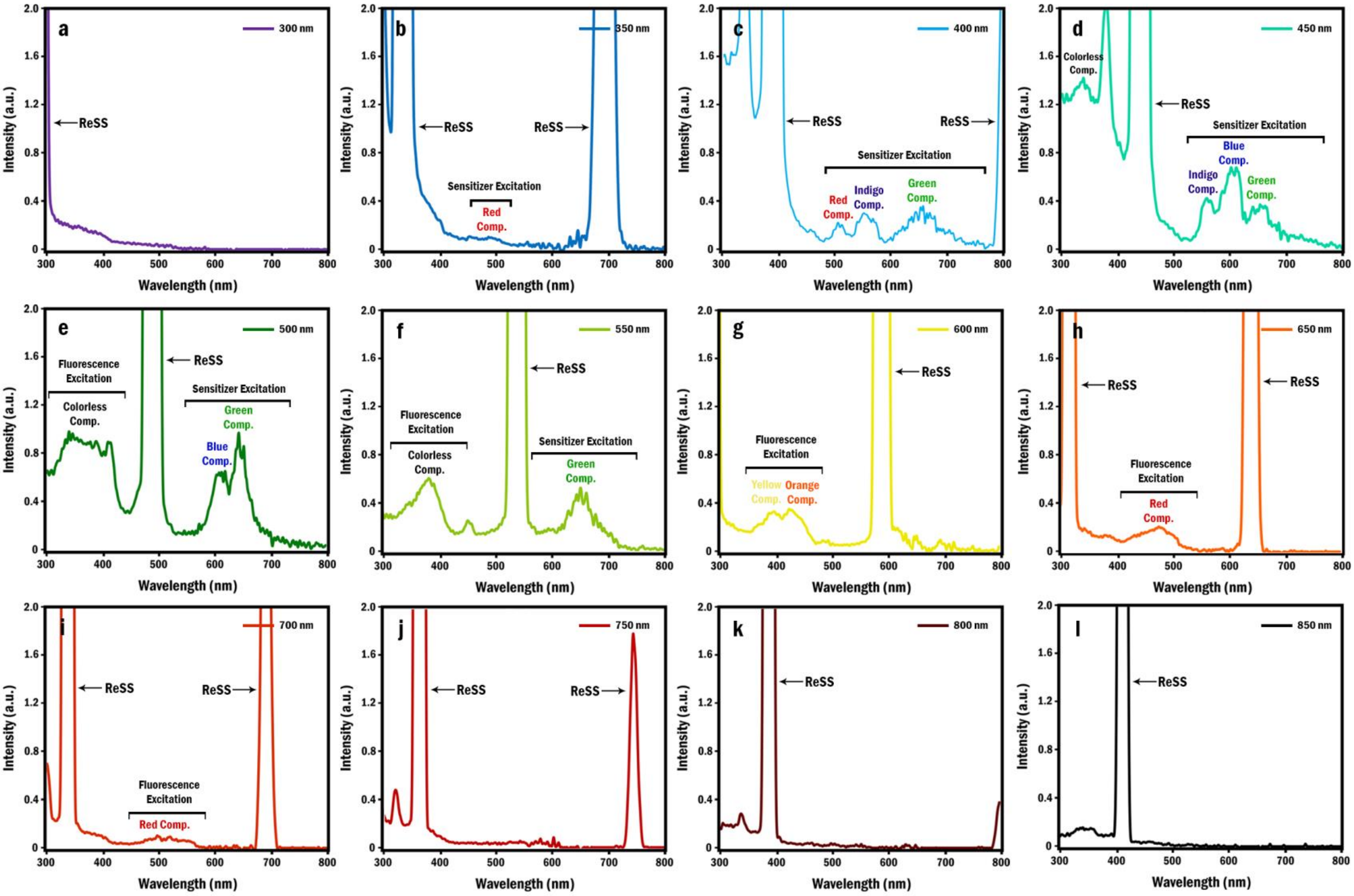

Figure S7. (a-l) Excitation spectra of NOG fibers in neutral aqueous medium in the region of 300-800 nm for several consecutive emission wavelengths

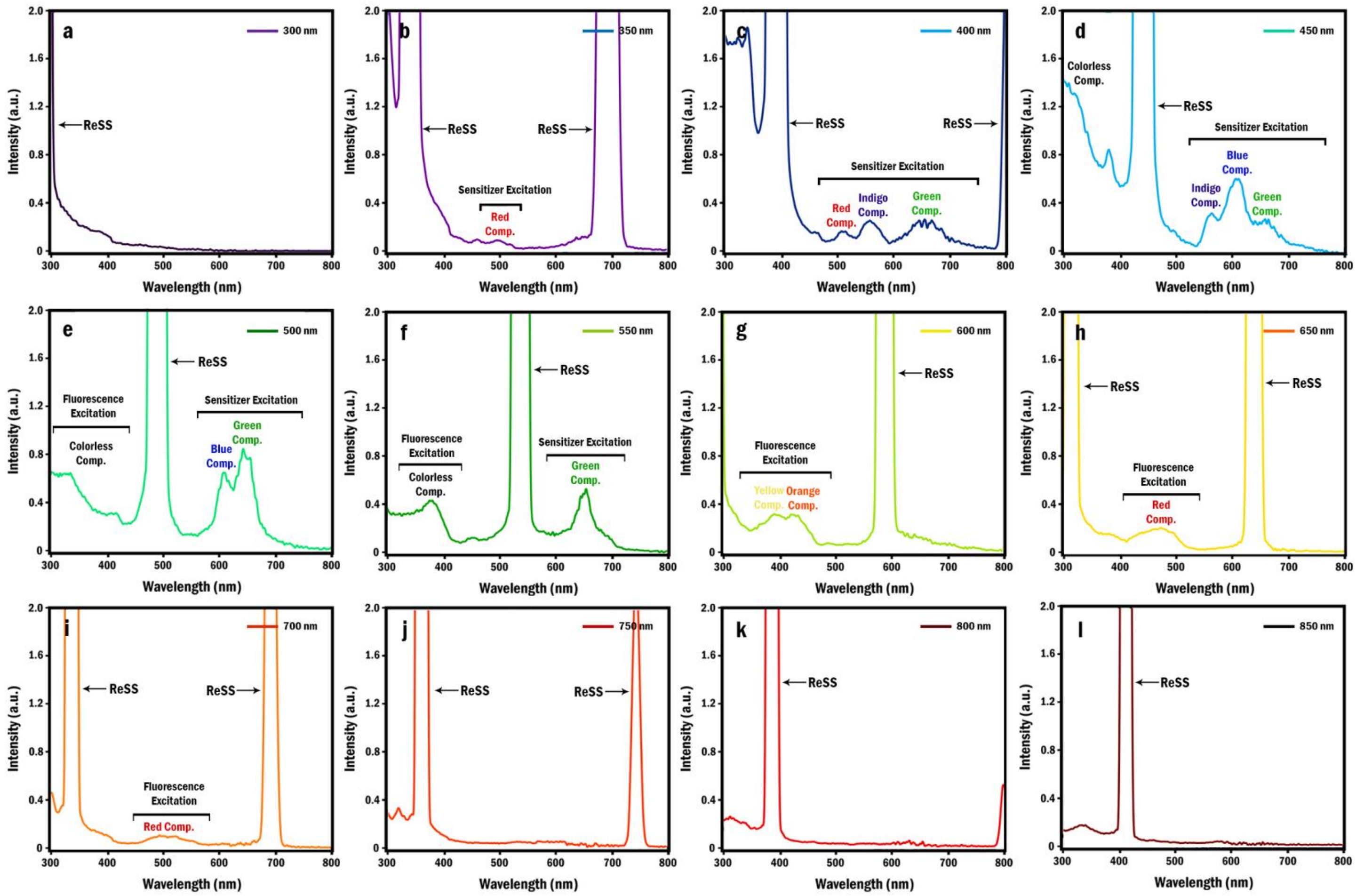

Figure S8. (a-l) Excitation spectra of NOG nanosheets in neutral aqueous medium in the region of 300-800 nm for several consecutive emission wavelengths
